# Supplementary material for: Reliable Characterization of Organic & Pharmaceutical Compounds with High Resolution Monochromated EEL Spectroscopy
Source: Polymers (Basel). 2020 Jun 27;12(7):1434. doi: 10.3390/polym12071434 (PMC7408036; doi:10.3390/polym12071434)
Supplement: Supplementary file 1 [file polymers-12-01434-s001.pdf]

Supplementary Materials

Samples

1,2,3,4,5,6 Cyclohexane-Hexacarboxylic acid mono-hydrate (Chemical Formula $C_{12}H_{12}O_{12}$, Molecular weight 348.22 g/mol) sample was purchased from (Aldrich), beta – Cyclodextrine ($C_{42}H_{70}O_{35}$, Molecular weight 1134.98 g/mol) was purchased from Acros Organics and tannic acid ($C_{76}H_{52}O_{46}$, Molecular weight 1701.20 g/mol) was purchased from sigma Aldirch.. The two peptides (pGlu-Cys(Trt)-Gly (TH_15 Chemical Formula $C_{29}H_{29}N_3O_5S$, Molecular weight 531,62 g/mol) and Tic-Cys-Aib (TH_27, Chemical Formula $C_{17}H_{23}N_3O_4S$, Molecular weight 365,45 g/mol) used in this study are Glutathione (GSH) structure-guided tripeptide analogues and they were designed and synthesized as substrates or inhibitors that could discriminate between allozymes of the MDR-involved human glutathione transferase P1-1 [50]. They were synthesized manually following the Fmoc/tBu strategy on -chlorotrityl resin (CTC) and purified (≥95%) by RP chromatography as described earlier by Zompra AA. et al [51]. All the samples are in amorphous form.

Piroxicam (Chemical Formula $C_{15}H_{13}N_3O_4S$, Molecular weight 331.348 g/mol) is used in the treatment of rheumatoid and osteoarthritis, primary dysmenorrhoea, postoperative pain; and act as an analgesic, especially where there is an inflammatory component. Two crystalline polymorphic form of Piroxicam (Form 1 and Form 2); (Form 1 Monoclinic Structure: a = 7.034 Å, b = 14.98940 Å, c = 13.89390 Å, α = 90°, β = 96.3870° γ = 90°; Form 2 Triclinic Structure: a = 10.34650 Å, b = 12.71260 Å, c = 12.80980 Å, α = 102.7800°, β = 99.9850° γ = 108.7280°) was used for the EELS study. Aripiprazole (Chemical Formula $C_{23}H_{27}Cl_2N_3O_2$ Molecular weight 448.385 g/mol) is used to prepare tablets to treat certain mental/mood disorders (such as bipolar disorder, schizophrenia, Tourette's syndrome, and irritability associated with autistic disorder). It may also be used in combination with other medication to treat depression. Aripiprazole is known as an antipsychotic drug (atypical type). It works by helping to restore the balance of certain natural chemicals in the brain (neurotransmitters). Two crystalline polymorphic form (Form II and Form IV) of Aripriprazole (Form II Orthorhombic Structure: a = 23.519 Å, b = 12.657 Å, c = 7.7560 Å, α = 90°, β = 90° γ = 90° ; Form IV Triclinic Structure: a = 8.5180 Å, b = 9.0350 Å, c = 30.417 Å, α = 88.072°, β = 86.550° γ = 73.874°) has been used for this study. Piroxicam and Aripriprazole

samples have been provided by Dr. Ulises Julio Amador Elizondo (Universidad CEU San Pablo, Spain) for the EELS work.

References

50. Zompra, A; Georgakis, N; Pappa, E; Thireou, T; Eliopoulos, E; Labrou, N; Cordopatis, P; Clonis, Y. Glutathione analogues as substrates or inhibitors that discriminate between allozymes of the MDR-involved human glutathione transferase P1-1. *Biopolymers*, **2016**, 106, 330.

51. Lloyd-Williams, P; Albericio, F.; Giralt, E. Chemical Approaches to the Synthesis of Peptides and Proteins, CRC, Boca Raton, FL **1997**.

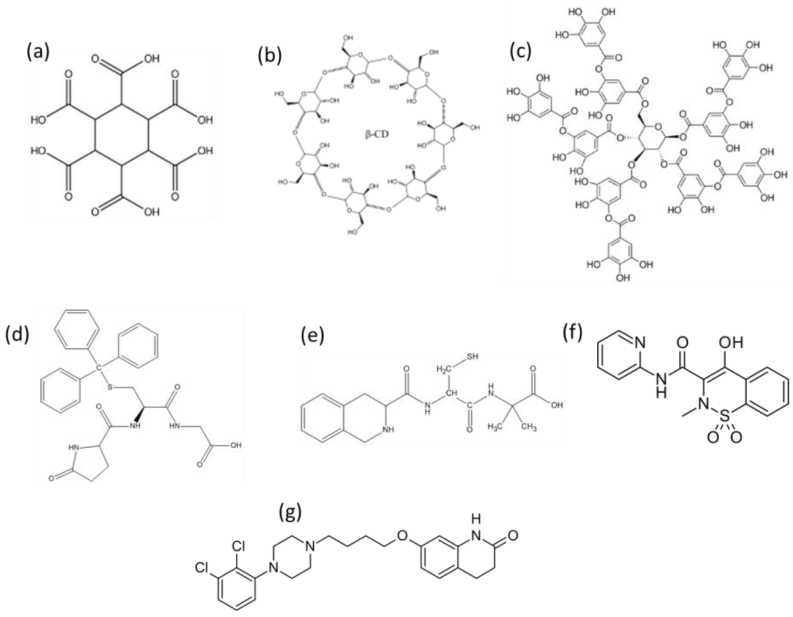

Figure S1: Molecular structure of Hexacarboxy Cyclohexane (a), Beta-cyclodextrin (b), Tannin (c), Peptide TH_15 (d) and Peptide TH_27 (e), Piroxicam (f), Aripiprazole (g).
